# Role of the gut microbiota in nutrient competition and protection against intestinal pathogen colonization

**DOI:** 10.1099/mic.0.001377

**Published:** 2023-08-04

**Authors:** Victoria Horrocks, Olivia G. King, Alexander Y. G. Yip, Inês Melo Marques, Julie A. K. McDonald

**Affiliations:** ^1^​ Centre for Bacterial Resistance Biology, Department of Life Sciences, Imperial College London, London SW7 2AZ, UK; ^2^​ Centre for Bacterial Resistance Biology, Department of Infectious Disease, Imperial College London, London SW7 2AZ, UK

**Keywords:** Gut microbiome, nutrient competition, colonisation resistance, antibiotics, pathogens

## Abstract

The human gut microbiota can restrict the growth of pathogens to prevent them from colonizing the intestine (‘colonization resistance’). However, antibiotic treatment can kill members of the gut microbiota (‘gut commensals’) and reduce competition for nutrients, making these nutrients available to support the growth of pathogens. This disturbance can lead to the growth and expansion of pathogens within the intestine (including antibiotic-resistant pathogens), where these pathogens can exploit the absence of competitors and the nutrient-enriched gut environment. In this review, we discuss nutrient competition between the gut microbiota and pathogens. We also provide an overview of how nutrient competition can be harnessed to support the design of next-generation microbiome therapeutics to restrict the growth of pathogens and prevent the development of invasive infections.

## Introduction

### What is the gut microbiota?

The human gut is colonized by a complex microbial community collectively referred to as the gut microbiota. The gut microbiota consists of a wide range of different micro-organisms, including bacteria, archaea, viruses, fungi and single-celled eukaryotes. The taxonomic composition of the gut microbiota changes throughout the gastrointestinal tract, according to nutrient availability, mucus structure, pH and oxygen availability [1–6]. In the adult faecal microbiota, 90 % of the commensal bacteria belong to the phyla Bacillota (formerly Firmicutes) and Bacteroidota (formerly Bacteroidetes), while the remaining 10 % belong to the phyla Actinomycetota (formerly Actinobacteria), Fusobacteriota (formerly Fusobacteria), Verrucomicrobiota (formerly Verrucomicrobia), and Pseudomonadota (formerly Proteobacteria) [7]. Of the bacteria within the adult gut microbiota, 99.9 % are obligate anaerobes and the remaining 0.1 % are facultative anaerobes [5, 8]. In infants, facultative anaerobes are more abundant, representing 30 % at 3 months, which decreases to just 1 % at 3 years [9].

### Functional redundancy in the gut microbiota

There is high inter-individual variation in the taxonomic composition of the gut microbiota, and no universal species are present in all healthy individuals [10]. Despite these considerable differences, there is significant overlap in the microbial functional genes present between individuals [10, 11]. For example, the Human Microbiome Project demonstrated that healthy individuals can have large differences in the taxonomic composition of their faecal microbiota but share similar functional gene profiles [10]. This is because the gut microbiota has a high degree of functional redundancy, where phylogenetically unrelated taxa contain genes that perform similar functions [12]. Functional redundancy allows the host to maintain a healthy gut microbiota to preserve stability and resilience in response to perturbations [13, 14]. Therefore, identifying which microbial species are present in the gut microbiota is not necessarily sufficient to determine the functional output of the gut microbiota. In addition to asking ‘Who is there?’ we must also ask ‘What are they doing?’ and ‘How are they doing it?’ to answer important mechanistic questions in microbiome research and develop new microbiome therapeutics.

### Role of the gut microbiota in the metabolism of dietary substrates

The gut microbiota plays a crucial role in the digestion of dietary and host substrates. The gut microbiota can metabolize dietary fibers that humans are otherwise unable to digest [15]. In the large intestine, the gut microbiota breaks down undigested fibers, such as resistant starch, cellulose, inulin and pectin, utilizing these nutrients as carbon sources to support their growth [15]. Many species within the gut microbiota possess carbohydrate-active enzymes, which are required to digest these dietary fibers into their constituent sugars (Table 1) [16]. Moreover, in the large intestine, the gut microbiota can also break down proteins to use as nitrogen sources to support their growth [17]. Host substrates, such as mucin and bile salts, can also be metabolized by the gut microbiota [18, 19].

**Table 1.** Dietary polysaccharides and their breakdown products

Polysaccharide	Degradation products	Reference
Starch	Glucose, maltose, trehalose	[130, 131]
Inulin	Fructose, glucose, sucrose	[132]
Pectin	Glucose, arabinose, galactose, xylose, mannose, fructose	[133]
Arabinogalactan	Arabinose, galactose	[134]
Mucin	N-acetylglucosamine, galactose, fucose, amino acids	[135]
Xylan	Xylose, arabinose	[136]
Cellulose	Glucose	[137]
β-glucan	Glucose	[137]
Xanthan	Glucose, mannose, glucuronic acid	[137]

The gut microbiota can also participate in cross-feeding, where metabolic intermediates (such as acetate, lactate, succinate and formate) that are produced by gut commensals can act as nutrients to support the growth of other gut commensals [20, 21]. For example, members of the Bacteroidota and Negativicutes can convert succinate into propionate [22, 23]. Multiple commensal species within Pseudomonadota and Bacillota, such as *Anaerobutyricum hallii *, *Desulfovibrio piger* and *Coprococcus catus *, can convert lactate to acetate, propionate and butyrate [22, 24–27]. In co-culture *A. hallii* could deplete lactate (produced from the fermentation of starch by *B. adolescentis *), which led to an increase in butyrate [25].

### Gut microbiome-mediated colonization resistance

The gut microbiota protects against intestinal colonization by pathogens via colonization resistance through both direct and indirect mechanisms. Gut microbiota with high diversity have higher colonization resistance than gut microbiota with low diversity as a result of antibiotic treatment [28]. Perturbation of the gut microbiome can disrupt colonization resistance leading to intestinal colonization by pathogens.

**Fig. 1.**
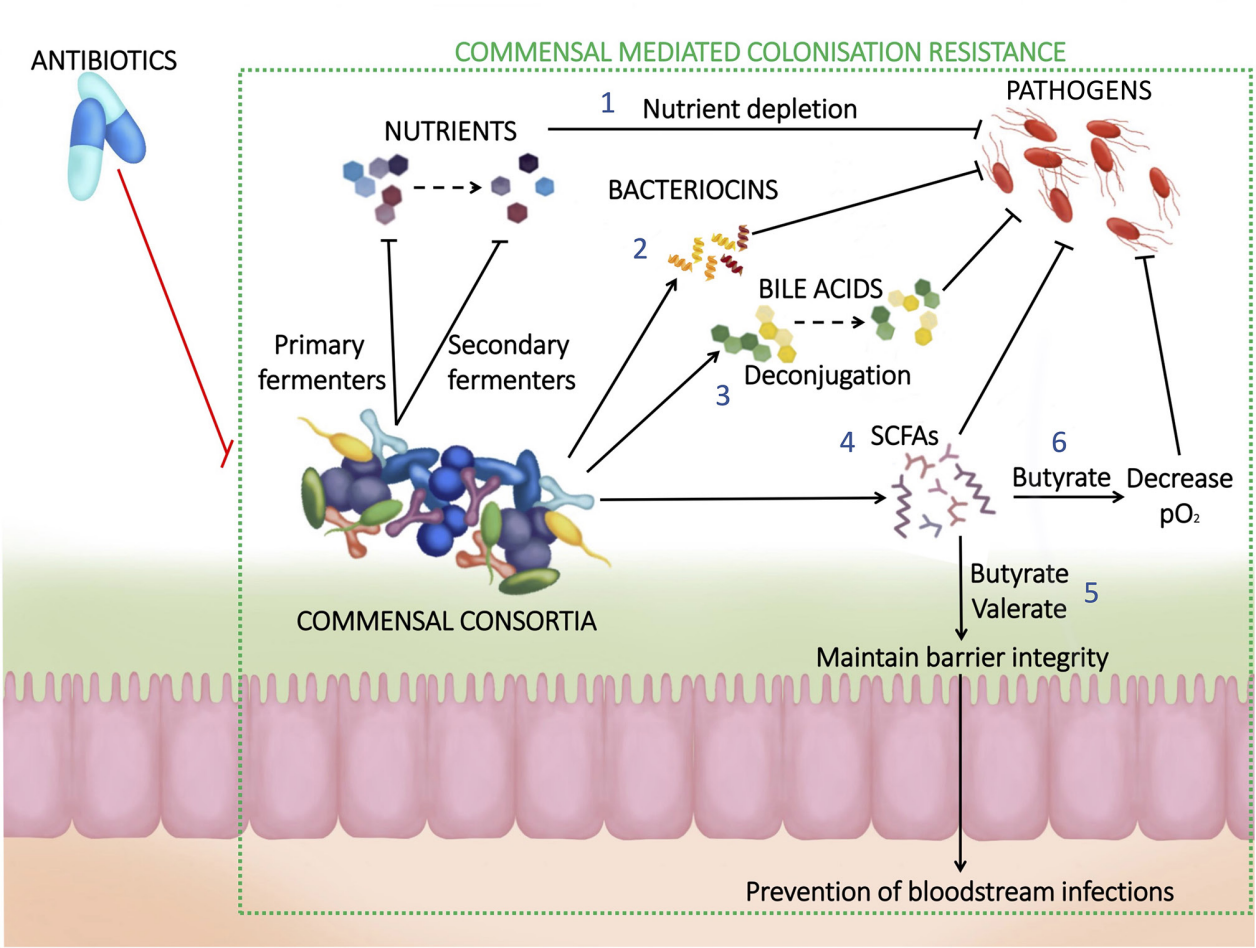
Commensal gut microbiota-mediated colonisation resistance. (1) Gut commensals compete with pathogens for nutrients that are essential to support their growth. (2) Some gut commensals produce small antimicrobial peptides (‘bacteriocins’), which can inhibit pathogen growth. (3) Metabolism of bile acids by gut commensals can reduce germination in spore-forming pathogens and inhibit vegetative growth. (4) Gut commensals produce SCFAs that inhibit pathogen growth through intracellular acidification. (5) Gut commensals produce butyrate and valerate, which maintain epithelial membrane barrier integrity and prevent pathogen translocation from the gut to the bloodstream. (6) Gut commensals produce butyrate, which is utilized by colonic epithelial cells as an energy source in a process that consumes oxygen. This creates an environment that is less supportive of the growth of facultatively anaerobic pathogens.

Antibiotic usage is widely documented to cause a substantial shift in the composition and functionality of the gut microbiota [29]. Antibiotic treatment – especially treatment with broad-spectrum antibiotics – kills members of the gut microbiota and disrupts colonization resistance (Fig. 1). This promotes intestinal colonization (and even domination) by pathogens such as *Clostridioides difficile *, carbapenem-resistant *Enterobacteriaceae* (CRE) and vancomycin-resistant *Enterococcus* (VRE) [30–32]. An improved understanding of colonization resistance mechanisms against pathogens is vital for the development of novel microbiome therapeutics to prevent or reduce pathogen intestinal colonization.

### Direct versus indirect mechanisms of colonization resistance

Mechanisms of colonization resistance can be divided into both direct and indirect mechanisms. Direct mechanisms of colonization involve bacteria–bacteria interactions that are independent of host involvement, while indirect mechanisms of colonization resistance involve bacteria–host interactions that rely on host involvement. Examples of direct mechanisms of colonization resistance include nutrient competition between gut commensals and pathogens, production of inhibitory metabolites by gut commensals, production of bacteriocins by gut commensals, and the presence of bacteriophages [33, 34]. Indirect mechanisms of colonization resistance include interactions between the gut microbiota and the host immune response, changes in the gut microbiota that influence oxygen availability, modulation of the mucus layer by the gut microbiota, and promotion of cytokine release by the gut microbiota [34]. This review will focus on discussing direct mechanisms of colonization resistance.

### Pathogen inhibition by short-chain fatty acids (SCFAs)

Metabolism by gut commensals drives colonization resistance not just by depleting nutrients, but also through the production of microbial metabolites. For example, the gut microbiota can ferment polysaccharides or proteins to produce short-chain fatty acids (SCFAs) [35]. Acetate, propionate and butyrate are the most abundant SCFAs in the gut, valerate is also present at lower concentrations [33, 36]. Microbial metabolites can inhibit the growth of some bacteria, including pathogens. For example, nonionized SCFAs can diffuse across the bacterial membrane and into the cytoplasm, where they dissociate into their ionized forms, lowering the intracellular pH and inhibiting the growth of susceptible bacteria, including antibiotic-resistant *Enterobacteriaceae* [37]. Administration of *Lactobacillus* to antibiotic-treated mice increased faecal butyrate levels and decreased intestinal colonization by *Klebsiella pneumoniae* [38]. Acetate production was associated with protection against *Escherichia coli* O157 in mice that were mono-colonized by *Bifidobacterium* [39]. Propionate produced by *Bacteroides thetaiotaomicron* negatively impacted the growth of *Salmonella enterica* serovar Typhimurium [40].

SCFA production can also reduce the pH of the intestine, which can influence the bacteria that can grow at that pH. For example, administering SCFA-producing commensals such as *Bifidobacterium longum* subspecies *infantis* to infants reduces intestinal pH from 5.97 to 5.15 and decreased virulence factor gene abundance [41–43]. Another study demonstrated that increasing the pH of *ex vivo* faecal cultures in a bioreactor system leads to an increase in *Enterobacteriacae* [44].

SCFAs also interact with the host to impact pathogen growth. Butyrate and valerate promote intestinal barrier function, which prevents the translocation of pathogens from the intestine into the bloodstream [45–48]. Butyrate acts as an energy source for colonic epithelial cells, which can improve gut barrier integrity [49]. In addition, butyrate interacts with gut epithelial cells to influence oxygen availability in the gut. When butyrate is reduced with antibiotic treatment oxygen availability increases, which promotes the growth of facultatively anaerobic pathogens [50].

### Bile metabolism

Other microbial metabolites can promote or inhibit the growth of some pathogens that colonize the intestine. Bile salts play an important role in promoting the germination of *C. difficile* spores or the inhibition of vegetative *C. difficile* cells [51]. Bile salt hydrolase is an enzyme produced by some gut commensals (such as members of the *Bacteroides, Bifidobacterium* and *Faecalibacterium* genera) that deconjugate conjugated primary bile acids, including taurocholate. Taurocholate promotes the germination of *C. difficile* spores, which leads to *C. difficile* colonization in the intestine [52]. Some gut commensals (such as *Clostridium scindens *) produce enzymes involved in the 7α-dehydroxylase pathway, which are involved in the conversion of unconjugated primary bile acids into secondary bile acids, such as deoxycholate and lithocholate. These secondary bile acids can inhibit vegetative *C. difficile* growth [53–55]. Additionally, while primary bile acids promote spore germination, secondary bile acids inhibit spore formation by *C. difficille* [56, 57]. Therefore, a healthy gut microbiota promotes colonization resistance against *C. difficile* through metabolism of bile acids to both reduce primary bile acids, which promote *C. difficile* spore germination, and increase secondary bile acids, which inhibit *C. difficile* vegetative growth.

### Nutrient competition

Nutrient availability significantly impacts the diversity and abundance of the micro-organisms that colonize the intestine [58–63]. Each gut commensal strain has its own nutrient utilization ability and preference, and competition is high between bacteria with overlapping nutrient utilization abilities [64, 65]. Pathogens must compete with gut commensals for nutrients to colonize the intestine, and pathogens can more easily colonize an intestine with a low diversity gut microbiota that does not utilize all the available nutrients (e.g. following antibiotic treatment). Bacteria compete for carbon and nitrogen sources that are essential to support their growth within the intestine [66–68]. However, bacteria can also compete for other compounds that support their growth, such as iron and zinc reservoirs [69, 70].

The nutrient niche theory was first described by Rolf Freter in 1983 and proposed that the composition of the gut microbiota is dictated by nutrient availability. It also proposed that a micro-organism will only colonize the intestine if it can utilize one or more limiting nutrients with greater efficiency than its competitors [71]. A deeper understanding of the diversity of nutrient sources used by both pathogens and gut commensals (and the metabolites produced through the metabolism of these nutrients) is crucial to understand how antibiotics promote intestinal colonization of pathogens, and how we can restore colonization resistance by using this knowledge to develop new microbiome therapeutics.

### Nutrient competition between similar bacterial taxa

Members of the same species have similar nutrient utilization profiles and therefore they are likely to occupy the same niche within the intestine. For example, Maldonado-Gómez and colleagues administered the probiotic strain *Bifidobacterium longum* AH1206 to healthy individuals and demonstrated that *B. longum* AH1206 was undetectable in the faeces from 64 % of individuals once administration was stopped, but that it persisted in the faeces of 27 % of individuals >166 days post-administration [72]. The baseline abundance of *B. longum* negatively correlated with the persistence of *B. longum* AH1206, suggesting these two *B. longum* strains occupied similar intestinal niches. Moreover, *B. longum* AH1206 persistence was associated with a reduced abundance of carbohydrate-utilizing genes detected in the probiotic-treated gut, suggesting that nutrient availability promoted colonization of this probiotic strain [72].

Another study by Lee *et al*. demonstrated that germ-free mice colonized by a single strain of *Bacteroides* species were resistant to colonization from the same, but not different, *Bacteroides* species [73]. The authors proposed that individual *Bacteroides* species occupy a unique niche within the intestine. They further discovered a class of polysaccharide utilisation loci (commensal colonization factors, or ‘ccf’), that was conserved amongst *Bacteroides* and required for colonization.

Several studies have demonstrated that commensal *E. coli* can compete with *E. coli* O157:H7 for nutrients. Maltby and colleagues demonstrated that two commensal *E. coli* strains (* E. coli* HS and *E. coli* Nissle 1917) could prevent *E. coli* O157:H7 intestinal colonization via nutrient competition [74]. *E. coli* O157:H7 could use the mucus-associated monosaccharides arabinose, galactose, N-acetylglucosamine, ribose and mannose to support its growth. *E. coli* HS could use arabinose, galactose, N-acetylglucosamine and ribose (but not mannose). *E. coli* Nissle 1917 could use arabinose, galactose, N-acetylglucosamine and mannose (but not ribose). *E. coli* HS and *E. coli* Nissle 1917 could not restrict *E. coli* O157:H7 growth individually as neither strain fully occupied the nutrient niche that was occupied by *E. coli* O157:H7. However, when *E. coli* O157:H7, *E. coli* HS and *E. coli* Nissle 1917 were grown together, *E. coli* HS and *E. coli* Nissle 1917 could use all five sugars that *E. coli* O157:H7 could use, and these two commensal strains could fully occupy the nutrient niche of *E. coli* O157:H7 to restrict its growth. In another study, Momose *et al*. demonstrated that commensal *E. coli* compete with *E. coli* O157:H7 for proline [75]. They showed that high-proline-utilising *E. coli* strains depleted the proline pool in germ-free mouse caecal contents and inhibited the growth of *E. coli* O157:H7, but this was reversed by adding excess proline.

Previous studies have also investigated nutrient competition between *Klebsiella oxytoca* and *Klebsiella michiganensis* (a member of the *K. oxytoca* complex) against antibiotic resistant *Enterobacteriaceae* [76, 77]. Oliveira and colleagues demonstrated that antibiotic-treated mice that were colonized with *K. michiganensis* were resistant to colonization with *E. coli* [77]. They also showed that antibiotic-treated mice that were administered galactitol (a nutrient that is utilised by *E. coli* but not *K. michiganensis *) eliminated colonization resistance mediated by *K. michiganensis *. This suggested that nutrient competition was the likely mechanism of colonization resistance conferred by *K. michiganensis *. In another study, Osbelt and colleagues screened faecal samples of healthy adults and children for their ability to inhibit multidrug-resistant *K. pneumoniae* growth in an *ex vivo* assay [76]. They found that *K. pneumoniae* growth varied by donor faecal microbiota, and that *K. oxytoca* was present at high levels in the two most protected faecal samples from children. They also showed that *K. oxytoca* accelerated clearance of *K. pneumoniae* in antibiotic-treated mice. Nutrient utilization assays demonstrated that *K. oxytoca* was able to utilize 100 carbon sources, while *K. pneumoniae* could only utilize 56 carbon sources, and there was an overlap in 55 of the carbon sources that *K. oxytoca* and *K. pneumoniae* could both utilize. Therefore, the authors suggested that the colonization resistance that *K. oxytoca* conferred against *K. pneumoniae* was due to competition for nutrients.

### Pathogen exploitation of the altered nutrient environment following antibiotic treatment to overcome colonization resistance

Antibiotics disrupt microbiota-mediated colonization resistance, promoting the colonization and expansion of pathogens within the intestine. Broad-spectrum antibiotics cause significant decreases in the abundance and diversity of a wide range of gut commensals, thus reducing competition for nutrients [6, 78]. These nutrient-defined intestinal niches can then be exploited by pathogens, such as *C. difficile *, CRE and *S*. Typhimurium [37, 38, 57, 79, 80].

Healthy gut microbiota can also restrict the growth of specific gut commensals as well. For example, commensal *Enterobacteriaceae* are typically found at low abundances in the healthy gut microbiota, composing just 0.1–1 % relative abundance on average [10]. However, in the faecal microbiota of a patient treated with amoxicillin-clavulanic acid, abundance of *Enterobacteriaceae* increased from 2–34 % after 4 days of antibiotic treatment [81].

According to the nutrient niche theory, *Enterobacteriaceae* growth is limited in the healthy gut microbiota due to competition for nutrient sources with other gut commensals. *Enterobacteriaceae* can also be suppressed by production of SCFA and other metabolites produced by gut commensals [39, 79, 82]. However, antibiotics disrupt colonization resistance and allow for the expansion of pathogens (Fig. 1) [83].

Antibiotic treatment can also lead to the overgrowth of multidrug-resistant (MDR) pathogens in the intestine [3, 84]. Dense intestinal colonization with these MDR pathogens can lead to the development of invasive infections (such as bloodstream infections or urinary tract infections) and can promote the transmission of these pathogens between patients — a particular problem for immunocompromised patients [85]. Restoring colonization resistance (or preventing the initial loss of colonization resistance) is vital to restrict the growth of MDR pathogens in the intestine following antibiotic treatment [86]. We recently demonstrated that broad-spectrum antibiotics (which promote intestinal colonization by CRE) altered the nutrient landscape in the gut by increasing the availability of various monosaccharides, disaccharides and amino acids [79]. These enriched nutrients were used as carbon and nitrogen sources to support CRE growth both *in vitro* and *in vivo*. We also demonstrated that CRE isolates had preferences for specific nutrients over others when presented with a mixture of the nutrients that were enriched with antibiotic treatment. Moreover, CRE isolates were able to grow to higher levels on these nutrients in the presence of oxygen, which is increased in the gut following antibiotic treatment.

Previous studies demonstrated that *C. difficile* can also use nutrients that are increased with antibiotic treatment to support its growth. Theriot and colleagues demonstrated that several nutrients (sorbitol, mannitol, arabitol, xylitol, gluconate, sucrose, lactate, raffinose, stachyose, galactose and fructose) were increased in the caecal contents of antibiotic-treated mice susceptible to intestinal colonization with *C. difficile* [32]. They demonstrated that *C. difficile* could utilize mannitol, fructose, sorbitol, raffinose and stachyose as carbon sources to support its growth. Another study by Fletcher and colleagues demonstrated that amino acids (in particular, proline and branched-chain amino acids) and carbohydrates were decreased in antibiotic-treated mouse caecal content over time in *C. difficile-*colonized mice [87]. Gene expression data was consistent with the finding that *C. difficile* used these nutrients to support its growth.

Pathogens can also utilize mucin-derived sugars to support their growth. Ng *et al*. demonstrated that *S*. Typhimurium and *C. difficile* expansion was aided by elevated sugars released from mucin in the caecal contents of antibiotic-treated mice [80]. *S*. Typhimurium was able to utilize sialic acid and fucose, and mutants deficient in these sugar utilization pathways had impaired intestinal colonization. *C. difficile* was able to utilize sialic acid, while a mutant that was unable to utilize sialic acid had impaired intestinal colonization. Hudson and colleagues demonstrated that *K. pneumoniae* was also able to utilize mucin-derived fucose to support its growth in the mouse intestine [88]. They found that a *K. pneumoniae* mutant, that was unable to metabolize fucose (*ΔfucI*), showed significantly decreased faecal shedding in mice and decreased growth in filtered cecal contents compared to wild-type *K. pneumoniae *.

Antibiotic treatment affects the redox state in the gut by reducing competition for electron acceptors, leading to blooms of *Enterobacteriaceae* [89] Oxygen and nitrate availability was increased in the intestine following antibiotic treatment due to a depletion of butyrate-producing bacteria [90, 91]. Depletion of butyrate switches the metabolism of intestinal epithelial cells from butyrate metabolism (which consumes oxygen) to glycolysis (which does not consume oxygen), leading to increased levels of oxygen in the intestine [92]. Nitrate, which can serve as an alternative electron acceptor to oxygen, is important for the survival of *E. coli* under anaerobic conditions and can allow *E. coli* to outcompete bacteria using fermentation for energy generation [93, 94]. Nitrate availability is increased in the intestine following antibiotic treatment or in an inflamed intestine [89, 94]. *S*. Typhimurium intestinal colonization can trigger an increase in nitrate by inducing inflammation and can exploit this mechanism for intestinal expansion [95]. However, commensal *E. coli* are able to compete with *S*. Typhimurium for nitrate to restrict its growth [96]. In a mouse model, probiotic *E. coli* Nissle 1917 engineered to be deficient in nitrate-respiration were less efficient at restricting *S*. Typhimurium growth than strains, which could utilize nitrate, demonstrating the importance of nutrient competition in maintaining colonization resistance [96].

### Harnessing nutrient competition to restrict intestinal growth by pathogens

**Fig. 2.**
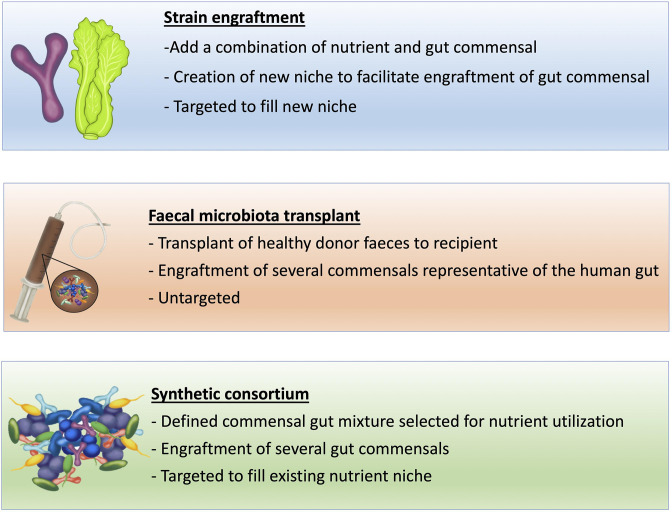
Methods to enhance the gut microbiome to restrict pathogen growth. Strain engraftment works through the creation of a new nutrient niche (introducing a new nutrient not utilized by other gut commensals) to allow engraftment of a target gut commensal strain by evading colonization resistance. Faecal microbiota transplant works by introducing an undefined mix of commensals to replenish the gut microbiota and restore colonization resistance. A synthetic consortium is designed to fill the existing nutrient niche to exclude a target pathogen.

**Fig. 3.**
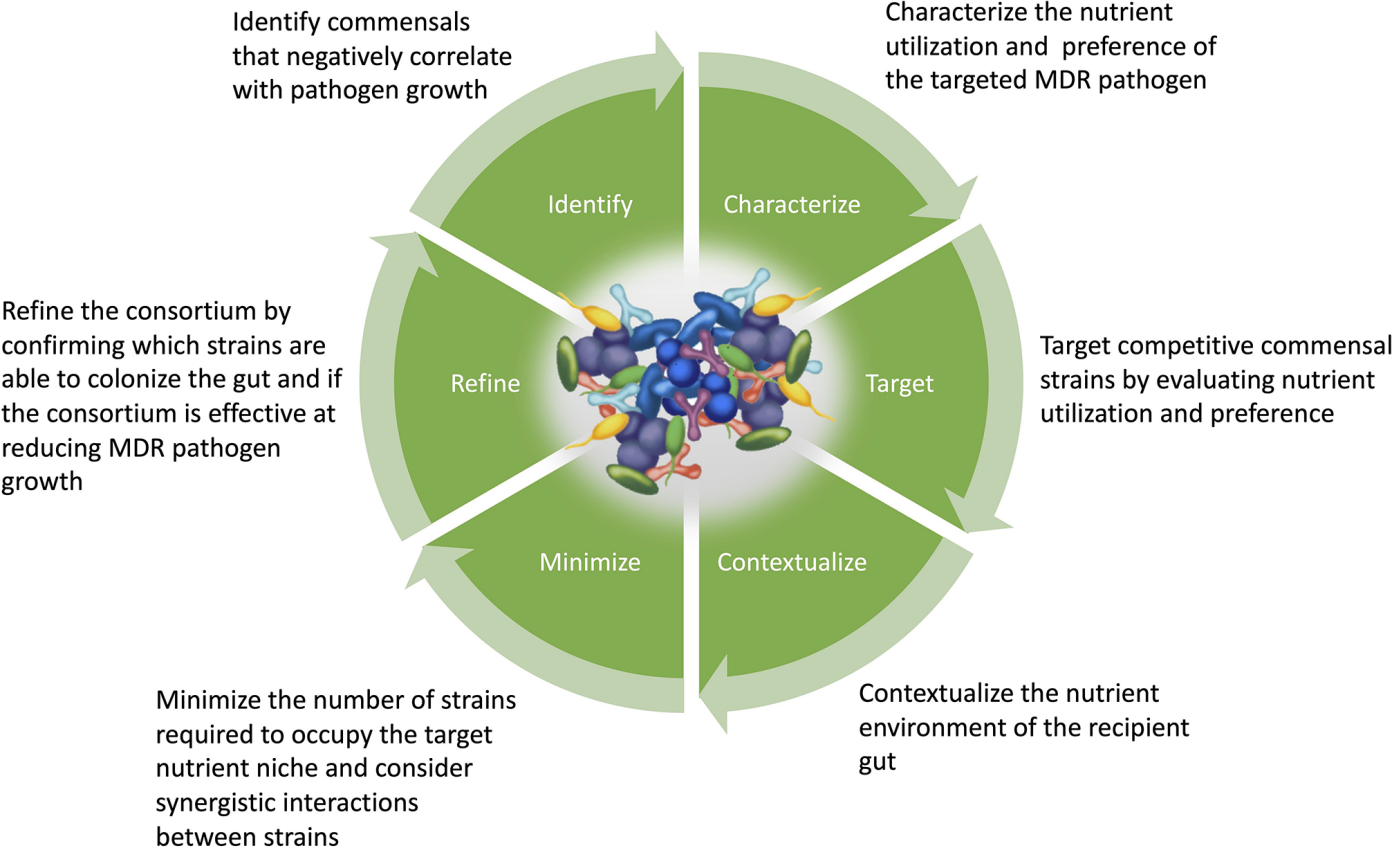
Stages in the design of a synthetic microbial consortium to target a specific pathogen.

We have demonstrated the important role that nutrient competition plays in gut microbiota-mediated colonization resistance against the intestinal colonization by pathogens. Using this information, new microbiome therapeutics need to be developed that modify or control the gut microbiota to re-establish colonization resistance through the restoration of nutrient competition (Figs 2 and 3).

Previous studies have demonstrated that it is possible to promote or restrict the growth of invading bacteria by altering the availability of nutrients, for example, by introducing nutrients to create a new nutrient-defined intestinal niche. In a study by Shepherd and colleagues, a *Bacteroides ovatus* strain was administered to human gut microbiota-associated mice with varying success depending on the donor microbiota [97]. This *B. ovatus* strain contained a rare porphyrin utilization locus, which allowed for the utilization of polysaccharides derived from seaweed. When mice were fed a diet high in seaweed after administering this *B. ovatus* strain*,* its abundance significantly increased, independent of the donor faecal microbiota [97]. This study demonstrated that the utilization of a specific carbon source can facilitate a bacterial strain to evade colonization resistance. Synergistic synbiotics have built upon a similar principle, as they contain a mixture of a substrate and a live micro-organism that can utilize that substrate to confer a health benefit to the host [98].

Diet can also influence pathogen growth in a context-dependent manner. For example, *B. thetaiotaomicron* was able to reduce intestinal growth of *Citrobacter rodentium* in mice fed a monosaccharide-rich diet, but not in a diet rich in monosaccharides and polysaccharides [99]. This was because *B. thetaiotaomicron* and *C. rodentium* competed for monosaccharides in the monosaccharide-only diet, but not in the monosaccharide and polysaccharide-rich diet that provided alternative nutrient sources for *B. thetaiotaomicron *, which could not be used by *C. rodentium *.

Faecal microbiota transplant (FMT) has been investigated to treat intestinal colonization with pathogens by reintroducing gut commensals into the intestine that are involved in both direct and indirect mechanisms of colonization resistance. For example, the success of FMT to treat *C. difficile* is associated with restoration of bile metabolism and SCFA production [100–106]. FMT has been used successfully to treat recurrent *C. difficile* infections with a higher success rate than antibiotic treatment [107]. *C. difficile *-associated diarrhoea was resolved in 81 % of patients treated with FMT compared to 31 % of patients treated with vancomycin alone [107]. A systematic review found that decolonization of MDR pathogens ranged from 20–90 % after FMT compared to between 11 and 66 % for controls [100]. A small trial found that FMT may be more successful at eradicating VRE carriage compared to CRE carriage [102]. However, FMT is not a risk-free procedure; whilst in most cases of FMT the adverse events are relatively minor, such as gastrointestinal discomfort [108], a much more serious consequence is the risk of transferring opportunistic pathogens, which may be carried asymptomatically by donors. Infections with Shiga toxin-producing *E. coli* and enteropathogenic *E. coli* have been reported following FMT pathogen screening, which impacts the viability of FMT by increasing costs and reducing the donor pool [109]. To bypass these issues, next-generation microbiome therapeutics should be donor-independent and contain a known composition of beneficial bacteria.

Previous work has demonstrated that synthetic microbial consortia (defined mixtures of gut commensals) can be effective at restricting the growth of pathogens in the intestine. Six phylogenetically distinct gut commensal strains were able to eradicate *C. difficile* colonization in mice [110]. A consortium of 33 strains isolated from human stool samples were used to prevent systemic infection of *S*. Typhimurium in antibiotic-treated mice [111]. The same consortium has been used to successfully eradicate *C. difficile* in two patients previously treated with multiple rounds of antibiotics, although there were differences in which commensal strains persisted in the faeces of the two patients following treatment [112].

The design of new microbiome therapeutics to restrict pathogen growth in the intestine requires the careful selection of gut commensals that are effective at restoring colonization resistance to promote pathogen clearance. One approach is to identify gut commensals that negatively correlate with pathogen growth. Isaac *et al*. treated mice with antibiotics with different spectra of activity and measured the amount of VRE in faecal samples [113]. Spearman correlation analysis was used to identify specific bacterial taxa that negatively correlated with VRE colonization. A synthetic bacterial consortium was developed consisting of strains from the genera *Alistipes *, *Barnesiella *, *Olsenella *, *Oscillibacter* and unclassified *Ruminococcaceae* that were isolated from mouse caecal content. This synthetic bacterial consortium restricted VRE intestinal colonization in antibiotic-treated mice. The effectiveness of this consortium was found to be due to depletion of fructose. *Olsenella* was capable of fructose utilization and reduced VRE colonization in mice both as a pre-treatment and when administered after VRE colonization [113]. Despite the restoration of colonization resistance being largely linked to *Olsenella* competing for fructose, the inhibition of VRE was greater with the whole consortium. This suggests that multiple mechanisms – and potential unidentified symbiotic interactions between members of the consortium – work together to reduce colonization of VRE.

Rational design of a microbial consortium can also be achieved by designing a mixture of commensals that targets the disruption of nutrient utilisation. As highlighted previously *C. difficile* is known to utilize host sugars following antibiotic treatment [80]. Pereira and colleagues designed a synthetic bacterial consortium that was capable of depleting N-acetylneuraminic acid and N-acetylglucosamine (mucus-derived sugars) that consisted of *Akkermansia muciniphila *, *Ruthenibacterium lactatiformans *, *Alistipes timonensis *, *Muribaculum intestinale *, and a *Bacteroides* sp. [114]. Depletion of N-acetylneuraminic acid and N-acetylglucosamine using this bacterial consortium restricted *C. difficile* growth both *in vitro* and *in vivo*.

Strategies to restore colonization resistance following antibiotic treatment should focus on introducing the minimal number of species possible to fill any unoccupied nutrient-defined intestinal niches to restrict pathogen growth. Due to the functional redundancy of the gut microbiota, it is possible to design a microbiome therapeutic that exhibits all the requisite functions that are required to have a therapeutic effect without administering a full and complex faecal microbiota. Defined microbial consortia must undergo regulatory approval, and the more complex the microbiome therapeutic, the more difficult it may be to pass this regulatory hurdle. However, no commensal strain is able to simultaneously use all nutrients which may be encountered in an antibiotic-treated gut microbiota [115, 116]. *Bifidobacterium* are present in almost all healthy human faecal samples and have genes that are predicted to be required for the degradation and internalization of a wide array of simple and complex carbohydrates [117–119]. Of the 47 sequenced *Bifidobacterium* strains investigated by Milani *et al.*, all strains could ferment glucose, sucrose and raffinose. However, the fermentation capabilities of other sugars (such as lactose, galactose, maltose, melibiose, fructose, lactulose, maltodextrins, turanose, β-gentibiose and xylose) varied for most strains that were tested [117]. *Bacteroides* are primary fermenters, which break down complex dietary and host carbohydrates into monosaccharides and can ferment these to SCFAs and other metabolites [120]. *Bacteroides* exhibit substantial variation in the degredation profile of polysaccharides [121]. *Bifidobacteria* are secondary fermenters, which utilize the products of primary fermentation to produce SCFA [122]. A combination of both primary and secondary fermenters would be required for a minimal consortium to cover major intestinal niches and to restore colonization resistance through the production of inhibitory metabolites.

Previous studies have demonstrated that gut commensal strains can provide colonization resistance against intestinal pathogens in a context-dependent manner. Eberl and colleagues demonstrated that gnotobiotic mice colonized by 12 murine gut commensals (OMM) [12] and commensal *E. coli* Mt1B1 prevented *S.* Typhimurium intestinal colonization [123]. However, gnotobiotic mice colonized with only three gut commensals and *E. coli* Mt1B1 were not protective. This study demonstrated that *E. coli* Mt1B1 colonization depleted galactitol in OMM [12] colonized mice, and that galactitol supported *S*. Typhimurium growth in OMM [12] colonized mice that lacked *E. coli* Mt1B1. This study also demonstrated that *Lachnospiraceae* contributed to colonization resistance against *S*. Typhimurium by consuming C5 and C6 sugars. Therefore, when developing microbiome therapeutics, it is important to study nutrient competition in the context of a gut microbiome that is representative of patients that will receive this therapeutic to properly assess its effectiveness.

When designing synthetic microbial consortia, it is important to consider synergistic interactions between different species within the consortium, as gut commensals act together to alter the gut environment [124]. This is especially important when designing synthetic microbial consortia that target nutrient competition, as gut commensals work together to fully degrade food within the intestine [125, 126]. For example, Caballero and colleagues demonstrated that a synthetic bacterial consortium containing *Blautia producta* and *Clostridium bolteae* restricts VRE intestinal colonisation in mice [127]. They demonstrated that *C. bolteae* did not directly restrict VRE intestinal colonisation, but rather enabled intestinal colonization with *B. producta *, which directly restricted VRE intestinal colonization. Another study, by Djukovic and colleagues, established that *Lactobacillus* species promoted the recovery of Eubacteriales and an overall increase in microbiota density following antibiotic treatment in mice [38]. The increase in Eubacteriales was associated with an increase of SCFA, notably butyrate, which was inhibitory to multidrug-resistant *Enterobacteriaceae.*

It is also important to consider that there may be differences in the preferences that bacteria have for particular available nutrients, which will impact the development of microbiome therapeutics. Different species of *Enterobacteriaceae* have different nutrient utilization profiles in aerobic and anaerobic environments and also have a different order of nutrient preference [79, 128]. These results suggest that microbiome therapeutics should include multiple gut commensals that are able to outcompete pathogens for all available nutrients that could support pathogen growth to fully occupy all available nutrient niches in the gut and effectively restore colonization resistance. If nutrients available in the antibiotic-treated gut microbiota are only partially depleted, then the microbiome therapeutic will be ineffective as the pathogen could switch to utilizing alternative available nutrients to support its growth.

## Conclusions

Exogenous micro-organisms can only colonize the intestine if they can occupy an available nutrient niche. The healthy gut microbiota fully occupies these niches and prevents pathogen intestinal colonization through multiple mechanisms, including nutrient competition. Antibiotic treatment causes significant disruption to the gut microbiota, which leads to the creation of new nutrient niches, which can become occupied by pathogens. With the urgent threat of antibiotic-resistant bacteria and the limitations of current treatment options, the development of novel microbiome therapeutics is vital to prevent this colonization or to promote decolonization, through introducing gut commensals that can outcompete pathogens for intestinal nutrients.

For microbiome therapeutics to be effective, a greater knowledge of certain areas of nutrient competition is required. Firstly, we must gain a better understanding of nutrient utilization and preferences by both pathogens and gut commensals. An improved understanding of how colonization resistance affects gut commensal colonization is essential to ensure the engraftment and persistence of a microbiome therapeutic. Further, knowledge of cross-feeding relationships is important to ensure the selected commensals can co-exist with commensals in the recipient’s gut microbiota. Development of microbiome therapeutics is also complicated by differences in the response of the gut microbiota to antibiotic treatment in different individuals due to differences in the composition and antibiotic resistance profiles of their baseline gut microbiota [129]. Individual bacterial taxa can show variable responses to antibiotics, which will impact the nutrient niches that are created in the intestine following antibiotic treatment [6]. Future studies should investigate how different antibiotics affect the nutrient and metabolite landscape encountered by pathogens in the intestine to inform selection of gut commensal strains that can occupy the available niches.
